# *Staphylococcus aureus* Shifts toward Commensalism in Response to *Corynebacterium* Species

**DOI:** 10.3389/fmicb.2016.01230

**Published:** 2016-08-17

**Authors:** Matthew M. Ramsey, Marcelo O. Freire, Rebecca A. Gabrilska, Kendra P. Rumbaugh, Katherine P. Lemon

**Affiliations:** ^1^Department of Microbiology, The Forsyth Institute, Cambridge, MAUSA; ^2^Department of Oral Medicine, Infection, and Immunity, Harvard School of Dental Medicine, Boston, MAUSA; ^3^Department of Applied Oral Sciences, The Forsyth Institute, Cambridge, MAUSA; ^4^Department of Surgery, Texas Tech University Health Sciences Center, Lubbock, TXUSA; ^5^Division of Infectious Diseases, Boston Children’s Hospital, Harvard Medical School, Boston, MAUSA

**Keywords:** quorum sensing (QS), *Corynebacterium*, *Staphylococcus aureus*, *agr* system, microbiome, commensal bacteria

## Abstract

*Staphylococcus aureus*–human interactions result in a continuum of outcomes from commensalism to pathogenesis. *S. aureus* is a clinically important pathogen that asymptomatically colonizes ~25% of humans as a member of the nostril and skin microbiota, where it resides with other bacteria including commensal *Corynebacterium* species. Commensal *Corynebacterium* spp. are also positively correlated with *S. aureus* in chronic polymicrobial diabetic foot infections, distinct from acute monomicrobial *S. aureus* infections. Recent work by our lab and others indicates that microbe–microbe interactions between *S. aureus* and human skin/nasal commensals, including *Corynebacterium* species, affect *S. aureus* behavior and fitness. Thus, we hypothesized that *S. aureus* interactions with *Corynebacterium* spp. diminish *S. aureus* virulence. We tested this by assaying for changes in *S. aureus* gene expression during *in vitro* mono- versus coculture with *Corynebacterium striatum*, a common skin and nasal commensal. We observed a broad shift in *S. aureus* gene transcription during *in vitro* growth with *C. striatum*, including increased transcription of genes known to exhibit increased expression during human nasal colonization and decreased transcription of virulence genes. *S. aureus* uses several regulatory pathways to transition between commensal and pathogenic states. One of these, the quorum signal accessory gene regulator (*agr*) system, was strongly inhibited in response to *Corynebacterium* spp. Phenotypically, *S. aureus* exposed to *C. striatum* exhibited increased adhesion to epithelial cells, reflecting a commensal state, and decreased hemolysin activity, reflecting an attenuation of virulence. Consistent with this, *S. aureus* displayed diminished fitness in experimental *in vivo* coinfection with *C. striatum* when compared to monoinfection. These data support a model in which *S. aureus* shifts from virulence toward a commensal state when exposed to commensal *Corynebacterium* species.

## Introduction

The bacterium *Staphylococcus aureus* is a common member of the human microbiota on the skin of the nasal vestibules (nostrils), where it colonizes more than a quarter of the U.S. population (Gorwitz et al., 2008), as well as on other skin surfaces. *S. aureus* is also a common human pathogen that causes a range of diseases from mild skin infections to lethal bacteremias (Lowy, 1998). *S. aureus* nostril colonization correlates with an increased risk of *S. aureus* infection (Wertheim et al., 2005) and approximately 80% of bloodstream infection isolates match nostril strains (Wertheim et al., 2004). In the past decade, methicillin-resistant *S. aureus* (MRSA) has emerged as an important public health issue; from 2005 to 2013, MRSA was responsible for nearly 10,000 deaths annually in the United States (CDC, 2005–2013). The possibility that *S. aureus* might acquire or evolve resistance to antibiotics beyond β-lactams, such as methicillin, is a grave concern in medicine and public health. This underlies the urgent need for research on novel antimicrobial (Conlon et al., 2013) and antivirulence therapies (Murray et al., 2014; Nielsen et al., 2014; Sully et al., 2014). Specific mechanisms of virulence in *S. aureus* have been studied for decades and are well characterized. Yet factors that influence the maintenance of harmless colonization (commensalism) and the transition from commensalism to virulence are still being defined.

*Staphylococcus aureus* possesses a broad array of colonization and virulence factors that interact with the human host; these include cytolysins, macromolecule degrading enzymes and immune evasion machinery (Lowy, 1998; Otto, 2010). *S. aureus* virulence is heavily affected by expression of the quorum sensing-controlled accessory gene regulator (*agr*) genetic locus, which has been studied extensively (for reviews Novick and Geisinger, 2008; Thoendel et al., 2011). The *agr* locus is divided into two divergent transcripts, RNAII and RNAIII, which comprise the *agrBDCA* operon and RNAIII regulatory RNA, respectively. The genes of the *agrBDCA* operon encode AgrB, which processes and exports an autoinducing peptide signal (AIP) derived from AgrD; and the AgrC sensor kinase with its cognate response regulator AgrA, which, when activated at high cell density, induces RNAII and RNAIII expression. Increased RNAIII transcription ultimately leads to the repression of adhesins and other surface proteins and the induction of capsule synthesis, toxins, proteases and other extracellular virulence factor production. Thus, *agr* activation is postulated to play a key role in *S. aureus’* transition from an adherent commensal lifestyle to an invasive pathogenic lifestyle (Novick and Geisinger, 2008; Thoendel et al., 2011).

As a member of the healthy skin microbiota, *S. aureus* interacts with a diverse array of other bacterial constituents; e.g., *S. aureus* primarily colonizes the nostrils (a.k.a. anterior nares) where it is detected in conjunction with members of the genera *Corynebacterium* and *Propionibacterium* (Uehara et al., 2000; Lina et al., 2003; Frank et al., 2010; Wos-Oxley et al., 2010; Oh et al., 2012; Yan et al., 2013). *S. aureus* also overlaps with other bacteria in various infection environments. For example, in chronic, polymicrobial diabetic foot infections (DFI) *S. aureus* is detected alongside numerous other bacterial species (Citron et al., 2007; Gardner et al., 2013); in particular, there is a positive correlation between *S. aureus* and *Corynebacterium* spp. in DFIs (Gardner et al., 2013). Recent work by us and others has begun to characterize specific microbe–microbe interactions of *S. aureus* with either *Propionibacterium* spp. (Wang et al., 2014; Wollenberg et al., 2014) or *Corynebacterium* spp. (Yan et al., 2013). We, and others, hypothesize that commensal bacteria play a role in maintaining health either by influencing *S. aureus* gene expression toward a commensal lifestyle or by limiting the expansion of *S. aureus*, both of which would limit the risk of acute infection.

In this study, we tested the hypothesis that *S. aureus* interactions with *Corynebacterium* spp. limit *S. aureus* virulence. Using a reductionist approach to mechanistically characterize interactions, we focused on *S. aureus* and *Corynebacterium striatum*, a skin commensal also commonly reported in DFIs (Citron et al., 2007). To assess how *S. aureus* responds to growth with *C. striatum*, we examined the *S. aureus* transcriptomes in mono- versus coculture *in vitro* with *C. striatum*. Growth with *C. striatum* resulted in global changes in *S. aureus* transcript abundance, including decreased expression of many genes induced by the *agr* QS system and increased expression of genes upregulated during *in vivo* nasal colonization (Burian et al., 2010a,b; Krismer et al., 2014). We found that exposure to cell-free conditioned medium (CFCM) from *C. striatum* and other *Corynebacterium* spp. was sufficient to decrease expression of an *agr*-induced promoter in *S. aureus.* When exposed to *C. striatum* CFCM, *S. aureus* displayed increased epithelial cell-adhesion activity and decreased hemolysin activity. In a murine subcutaneous abscess model, we observed that, compared to monoinfection, *S. aureus* abundance *in vivo* was diminished during coinfection with *C. striatum*, i.e., *S. aureus* was a less successful pathogen. These data demonstrate diminished virulence of *S. aureus* in response to commensal *Corynebacterium* spp., which is consistent with a shift to a commensal lifestyle when in the presence of a healthy microbiota rich with *Corynebacterium* spp. Overall, these results suggest a beneficial role for non-pathogenic members of the *Corynebacterium* genus in limiting *S. aureus* virulence.

## Materials and Methods

### Strains and Media

Strains used in this study are described in Supplementary Table S2. Unless indicated, *Staphylococcus* spp. and *Corynebacterium* spp. were routinely cultured using Brain Heart Infusion (BHI) broth or solid agar medium and *Escherichia coli* was grown on Lysogeny Broth (LB) medium. For indicated experiments, a chemically defined medium (CDM) (Brown and Whiteley, 2007) was used with the addition of 25 mM 2-(*N*-morpholino)ethanesulfonic acid (MES) buffer at pH 6 or pH 7.5. All cultures were grown at 37°C in standard atmosphere; liquid cultures were shaken at 250 RPM. Antibiotics were used at the following concentrations: ampicillin 100 μg/ml, erythromycin 10 μg/ml, tetracycline 3 μg/ml, fosfomycin 20 μg/ml and chloramphenicol 20 μg/ml for *E. coli* and 10 μg/ml for *S. aureus*. We selectively enumerated *S. aureus* on Mannitol Salts Agar (MSA) and *Corynebacterium* spp. on BHI agar with 20 μg/ml fosfomycin using colony-forming unit (CFU) measurement via track dilution as described previously (Jett et al., 1997).

### DNA and Plasmid Manipulations

DNA and plasmid isolation were performed using standard methods (Ausubel et al., 2002). Restriction endonucleases and DNA modification enzymes were purchased from New England Biolabs. Chromosomal DNA from all bacteria was isolated using DNeasy tissue kits (Qiagen); plasmid isolations were performed using QIAprep spin miniprep kits (Qiagen). For both kits, *S. aureus* cells were lysed by adding 25 μg of lysostaphin (Sigma–Aldrich) to the kit lysis buffer and incubating at 37°C for 30 m prior to following the manufacturer’s protocol. DNA fragments were purified using QIAquick mini-elute PCR purification kits, and PCR was performed using GoTaq Green (Promega). Primers used in this study are indicated in Supplementary Table S3. DNA sequencing was performed by automated sequencing technology through Macrogen Co. (Macrogen USA).

### Markerless Gene Deletion Construction in *S. aureus*

Markerless gene deletions were generated using a previously established protocol with derivatives of the temperature sensitive *S. aureus* shuttle vector pKFT (Kato and Sugai, 2011). Surface protein A (*spa*) deletion was accomplished using pLF048, a pKFT derivative (Foulston et al., 2014). pKFT-derivative vectors were electroporated into *S. aureus* RN4220 and grown at room temperature for 2 h before plating on BHI tetracycline 3 μg/ml agar medium and incubated at 30°C for ~36 h. Replicating plasmids were purified from *S. aureus* RN4220 prior to electroporation into *S. aureus* JE2 and treated as previously described (Kato and Sugai, 2011). Markerless tet^S^ mutants were confirmed by PCR using primers external to flanking regions of the pKFT derivatives (oKL377 and oKL378 for the *spa* deletion) followed by amplicon sequencing to verify loss of the targeted gene and religation of the flanking regions.

### Construction of the *spa*-Expression Vector

To express *spa in trans* in the Δ*spa* mutant, we cloned the *spa* gene with its native promoter region into pEPSA5 (Forsyth et al., 2002). Using primers oKL554 and oKL555, we amplified the P*_spa_*-*spa*-containing fragment, then digested it and pEPSA5 with SalI and BamHI (NEB). We electroporated pEPSA5 and pEPSA5-*spa* into *S. aureus* RN4220 then repurified and transformed each respectively into KPL2389 to generate the Δ*spa* mutant with the empty vector control (KPL2496) and *spa*-complemented strain (KPL 2497).

### Mono- and Coculture Assays

Colony cultures (Zheng and Stewart, 2002) were prepared similar to previously described on agarose medium (Ramsey and Whiteley, 2009). Briefly, CDM 1% agarose solid medium was prepared using 25 mM MES buffered CDM at pH 6.0. Sterile 25 mm 0.2 μm polycarbonate membranes (Millipore #GTTP02500) were placed on solid agarose medium and inoculated with 10 μl of liquid CDM containing either 5 × 10^6^ *C. striatum* or 1 × 10^4^ *S. aureus* separately in monoculture or combined in coculture. Inoculated spots were allowed to briefly air dry then transferred to 37°C for 18 h. Next, membranes were aseptically transferred to fresh medium and incubated at 37°C for another 4 h. To harvest cells, membranes were either immediately transferred to RNALater (Ambion) for RNAseq experiments (described below); transferred to 1 ml sterile Phosphate Buffered Saline (PBS), vortexed for 30 s and serially diluted for CFU enumeration; or transferred to 1 ml sterile PBS, vortexed for 30 s, pelleted by centrifugation at 10,000 *g* for 5 m then resuspended in 300 μl of lysis buffer for β-galactosidase assays (described below).

### RNA Isolation

Bacterial cells resuspended in 1 ml of RNALater (Ambion) (see above) were pelleted by centrifugation (10,000 *g* at 4°C for 10 m). Cell pellets were stored at -20°C. For lysis, cells were resuspended in 100 μL TE buffer with 10 mg/ml lysozyme and 25 μg/ml lysostaphin and incubated 20 m at 37°C. After adding 900 μl of RNA Bee (Amsbio), the mixture was transferred to 2 ml Lysing Matrix B bead tubes (MP Biomedicals) and bead beat in a FastPrep-24 (MP Biomedicals) 6x at the highest setting for 30 s each, with 30 s of rest on ice between each cycle. Next, 0.2 ml of chloroform was added to each tube and vortexed for 30 s then stored on ice for 5 m prior to centrifugation (12,000 *g* for 15 m at 4°C). The (upper) aqueous phase was removed and mixed with 0.5 ml of isopropanol in a microcentrifuge tube and incubated 10 m at room temperature (RT). After centrifugation at 12,000 *g* for 5 m at RT, the supernatant was carefully removed. One ml of ice-cold 75% ethanol was added to each tube, vortexed briefly and then centrifuged for 5 m at 7,500 *g*. This 75% ethanol wash was repeated, then RNA pellets were air dried at RT for 5–10 m. RNA was resuspended in 30 μl of nuclease-free ddH_2_O. DNA contamination was removed by digestion with 10 U of RQ1 DNAse (Promega) in a 100 μl reaction for 30 m at 37°C. RNA was then re-purified using RNABee as in the steps above. Final RNA samples were resuspended in 30 μl of nuclease-free H_2_O. RNA integrity was verified by visualization on a SYBR-Safe (Invitrogen) stained agarose gel and RNA quantity was determined by spectrophotometric analysis.

### RNAseq Library Preparation

RNAseq libraries were prepared using methods adapted from those described previously (Jorth et al., 2014). Ribosomal RNA was removed with the RiboZero Bacterial kit (Epicentre) per the manufacturer’s instructions. RNA was then fragmented with the NEBNext Magnesium RNA Fragmentation Module (NEB) and ethanol Na-acetate precipitated with the addition of 2 μl of linear acrylamide (NEB). Precipitated RNA was resuspended in 14 μl of nuclease-free H_2_O. Sequencing libraries of fragmented RNA were assembled using the NEBNext sRNA library prep kit (NEB) per the manufacturer’s instructions. Assembled libraries were run on 6% polyacrylamide TBE gels and bands between 130 and 300 bp were gel purified and extracted as per the NEBNext sRNA library kit instructions. Libraries were assayed for concentration and integrity on a Bioanalyzer 2100 (Agilent). RNAseq libraries were submitted to Tufts University Core Facility Genomics for sequencing using single-end 50 bp reads on an Illumina HiSeq 2500 system.

### RNAseq Results Validation

Separate quantification of gene expression from *S. aureus* JE2 was determined by quantitative Reverse Transcriptase PCR (qRT-PCR) from the original non-rRNA-depleted RNA samples and additional experimental samples generated under identical conditions. RNA preparation with DNA removal was performed as above and 0.5 ng of total RNA was used for qRT-PCR with a qScript One-Step SYBR Green qRT-PCR kit (Quanta Biosciences) to determine overall transcript abundance in each experimental condition. We used primers oKL311 and oKL312 to measure *spa* transcript abundance and oKL339 and oKL340 to measure *gyrB* abundance as an internal control. Controls lacking reverse transcriptase were also performed to verify lack of contaminating DNA. Comparisons of relative abundance of *spa* expression normalized to *gyrB* expression per sample were used to calculate fold change of *spa* expression and compared to RNAseq data under the same conditions (**Table 1**).

**Table 1:** Transcriptional response of *S. aureus* to cocultivation with *C. striatum*.

^a^Fold change	*q*-value	Gene name	ORF#	Protein product
260.5	0	*spa*	^b^0113	IgG binding protein A
10.0	3.9^-31^	*lrgB*	0257	Antiholin-like protein LrgB
7.0	2.3^-7^	*sarT*	2437	Accessory regulator T
7.0	9.3^-15^	*–*	1065	Exfoliative toxin A
5.0	6.7^-7^	*sbnC*	120	*lucC* siderophore biosynthesis protein
3.0	2.0^-3^	*metI*	360	Cys/Met biosynthesis enzyme
2.9	7.4^-6^	*oatA*	2503	Secretory antigen precursor *SsaA*
2.6	2.9^-6^	*clfB*	2565	Clumping factor b
2.1	3.1^-3^	*isdA*	1029	Iron transport associated domain-containing protein
-54.3	0	*psmβ1*	1067	Phenol soluble modulin β1
-16.8	2.5^-179^	*psmβ2*	1068	Pphenol soluble modulin β2
-8.3	5.3^-10^	*hlb*	1918	Truncated β-hemolysin / phospholipase C
-8.2	9.0^-71^	*–*	1988	δ-hemolysin
-2.7	2.4^-5^	*agrB*	^c^1989	Accessory gene regulator protein B
-2.7	1.9^-5^	*agrA*	^c^1992	Accessory gene regulator protein A
-2.5	2.0^-5^	*agrD*	^c^1990	Accessory gene regulator protein D
-2.0	2.3^-4^	*agrC*	^c^1991	Accessory gene regulator protein C

*This is a select set of the 469 *S. aureus* genes (Supplementary Table S1) that were differentially expressed ≥2-fold in coculture with *C. striatum*. ^a^Data are presented as fold change in coculture compared to monoculture assessed from biological duplicates. All genes included exhibited differential expression with a significance of *q* < 0.01 using a Benjamini–Hochberg correction with a false discovery rate <1%. ^b^When measured by qRT-Realtime-PCR, SAUSA300_0113 (*spa*) transcript level increased 186.6 ± 60.9-fold when calculated with *gyrB* (SAUSA300_0005) as a non-variable expression control between conditions. ^c^Denotes ORFs identified as part of the *agr* locus.*

### RNAseq Data Analysis

The *S. aureus* USA300 FPR3757 genome sequence (Diep et al., 2006) (NCBI accession #NC_007793) was used for RNAseq read alignment. FASTQ files were aligned to the *S. aureus* JE2 (NC_007793) genome using Rockhopper (McClure et al., 2013) with default settings.

### Nucleotide Sequence Accession Numbers

RNAseq sequencing data are available at NCBI in the sequence read archive under BioProject accession number PRJNA335565.

### AIP-1 Preparation

AIP-1 production was performed similarly to methods described previously (Thoendel and Horswill, 2009). Briefly, *E. coli* strain AH594 carrying an arabinose-inducible AIP-1 synthesizing gene locus (P_BAD_-*agrBD*) was grown overnight at 37°C shaking at 200 RPM in 5 ml of LB with 100 μg/ml ampicillin. A 1:50 dilution of overnight culture was added to 50 ml LB without ampicillin in a 250 ml flask and was shaken at 200 RPM incubated at 37°C for 90 min prior to addition of arabinose to a final concentration of 0.02%. The induced culture was further incubated in the same conditions for 4 h. Next the cell culture was transferred to a sterile 50 ml conical centrifuge tube and cell supernatants were removed after centrifugation at 7,000 *g* for 10 m at 4°C. Cell supernatants were next passed through a 0.2 μm filter (Millipore, #SCGP00525) to remove remaining cell debris and then passed through a 3 kDa-cutoff size exclusion filter (Millipore #UFC900324). Filtrates were prepared for storage by addition of sterile glycerol and dithiothreitol to concentrations of 10% and 1 mM respectively. Aliquots of 1.5 ml each were stored at -80°C and exhibited full activity for >30 days.

### *Corynebacterium* spp. CFCM Preparation

Cell-free conditioned medium was generated from each indicated *Corynebacterium* spp. after inoculation into BHI cultures and growth at 37°C shaking at 200 RPM for 48 h. Conditioned medium was removed after centrifugation of 48-h cultures for 10 m at 7,000 *g* and then passed through a 0.2 μm filter (Millipore, #SCGP00525) yielding CFCM, which was either used directly for experiments, frozen at -80°C prior to use or further passed through a 3 kDa molecular weight size exclusion filter (Millipore #UFC900324) then frozen at -80°C. For some samples, CFCM was heat treated at 95°C for 15 m. For other samples, CFCM was Proteinase K (Promega #V3021) treated by addition of 100 μg in 1 ml of CFCM prior to incubation at 55°C for 1 h followed by a 95°C heat inactivation for 10 m prior to testing for the effect on AIP-1.

### *S. aureus* AIP-1 Luminescence Detection Assays

AIP-1-dependent luminescence assays were performed similarly to those described previously (Jensen et al., 2008; Thoendel and Horswill, 2009). Briefly, 100 μl of an overnight culture of the AIP-1-responsive *agrP3-lux* luminescence reporter strain ROJ143 grown in 3 ml BHI with 10 μg/ml chloramphenicol was inoculated into 5 ml BHI with 10 μg/ml chloramphenicol and incubated at 37°C, shaking at 200 RPM for 1.5 h. Optical density at 600 nm (OD_600_) was measured and cells were then diluted in BHI to an OD_600_ of 0.1 and 100 μl were added to each well of a 96-well plate to be combined with mixes of CFCM for testing. Meanwhile, 150 μL of *E. coli* AIP-1-containing CFCM was incubated in a 1.5 ml microcentrifuge tube for 2 h at 37°C with an equal volume of one of the following: BHI or *Corynebacterium* spp. CFCM. Next, 25 μl of each incubated solution was added to four separate wells containing 100 μl of the ROJ143 reporter strain and incubated for 3 h at 37°C prior to reading luminescence on a BioTek Synergy HT illuminometer with a 0.5 s integration time. Luminescence was quantified as the average of four replicate wells each from 3 independent experiments.

### *S. aureus* Human Epithelial Cell Attachment Assay

We adapted this assay from a previously published protocol (Weidenmaier et al., 2004) and A549 cell cultures were performed identically with the exception that F-12K medium and 10% Fetal Bovine Serum (Life Technologies) was used. A549 cells were grown to 90–95% confluency (5 × 10^4^/well) in 24-well plates and washed 3x in serum-free F-12K medium before addition of *S. aureus*. We added a 1:10 dilution of *E. coli* CFCM containing AIP-1 into 1.5 ml cultures containing 1:100 inoculums from overnight cultures of either wild-type or *agrA*-deficient *S. aureus* JE2. A separate set of tubes was prepared identically plus the addition of a 1:2 dilution of 3 kDa-filtered *C. striatum* CFCM. These cultures were incubated at 37°C shaking at 200 RPM for 4 h. Late exponential cells were washed 3x in BHI and then diluted in serum-free 37°C F-12K medium and added to each well in 500 μl volumes at an MOI of 25. *S. aureus* cells from each growth condition and A549 cells were coincubated for 1h at 37°C and 5% CO_2_. Aliquots of planktonic cells were removed after incubation for CFU enumeration and A549 cell cultures were then washed 3x with sterile PBS. Five hundred microliter of PBS with 0.04% Triton-X100 was added to each well and incubated for 5 m at ambient temperature before epithelial cells were disrupted by vigorous micropipette aspiration. *S. aureus*-containing suspensions were transferred to microcentrifuge tubes and vortexed for 30 s each before being serially diluted for CFU enumeration. CFU values for attached cells were divided by their respective planktonic CFU values to determine % attachment for each condition. Percent attachment was divided by the % attachment for the WT *S. aureus* condition to determine fold change differences in attachment for each condition. Attachment was measured from technical triplicates for each condition across three biological replicates from separate A549 cell cultures.

### Immunofluorescence IgG-Capture Assay, Microscopy and Image Analysis

Cultures were grown as described in ‘mono- and coculture assays.’ Polycarbonate membranes were transferred to 1.5 ml microcentrifuge tubes containing 1 ml of ice-cold 95% methanol and stored at -20°C. Cells were removed from membranes by gentle agitation and membranes were discarded. We added 5 μl of cell suspension to poly-lysine coated slides (Corning). These were air dried for ~5 m. To block, 100 μl of PBS with 1% BSA was added to each sample and incubated for 1 h at RT. Blocking solution was removed by pipette after 1 h and an equal volume was added then removed immediately after. Fifty microliter of rabbit α-goat FITC-conjugated IgG antibody (Life Technologies) diluted 1:500 in PBS with 1% BSA was added to the sample and incubated in the dark at RT for 1 h. Antibody solution was removed by pipette and the sample was rinsed 3x with 200 μl PBS with 0.1% Triton X100 which was gently added and removed by pipette. The sample was rinsed 1x with 100 μl PBS and excess liquid was removed by pipette without allowing the sample to air dry. Seven microliter of DAPI mounting medium (Southern Biotech) was quickly added and the sample was covered with a 25 × 25 mm #1 coverslip and incubated in the dark for >10 m at RT.

Each sample was imaged at 1000x magnification on a Zeiss Axio Observer using manufacturers filter settings for DAPI and FITC acquisition and identical exposure times for all samples based on autofluorescence of a non-FITC stained control sample. Three randomly selected fields of view were taken of each sample from three biological replicates for each growth condition. Images were analyzed using ImageJ software (NIH). Due to pronounced differences in DAPI-staining intensity between species, *S. aureus* was easily quantified from coculture samples by thresholding images in the DAPI channel. Cells positively stained for FITC were also identified by thresholding based on a non-FITC stained control. Using identical thresholding settings, total *S. aureus* (DAPI) versus SpA-expressing *S. aureus* (FITC) were counted in all fields of view using the ‘Analyze Particles’ function.

### *S. aureus* Rabbit Erythrocyte Lysis Assay

This assay was adapted from a previously published protocol (Pang et al., 2010). We added a 1:10 dilution of *E. coli* CFCM containing AIP-1 into 1.5 ml cultures containing 1:100 inoculums of either wild-type or *agrA*-deficient *S. aureus* JE2. A separate set of tubes was prepared identically plus the addition of a 1:2 dilution of 3 kDa-filtered *C. striatum* CFCM. These cultures were incubated at 37°C shaking at 200 RPM for 4 h. After incubation, CFCM was generated from each *S. aureus* culture by passage through a 0.2 μm filter (Millipore, #SCGP00525). Rabbit blood (Hemostat Laboratories, USA) was diluted v/v to 3% in sterile PBS then 70 μl was combined with 30 μl of each *S. aureus* CFCM in eight replicates for each condition in a 96-well plate. Blood was also combined at the same ratio with BHI as a negative control and BHI was used as a blank for OD_630_ measurements. Plates were incubated at 37°C for 40 m then OD_630_ was read to determine rabbit erythrocyte lysis.

### *In vivo* Murine Abscess Growth

Murine abscesses were generated essentially as described previously (Mastropaolo et al., 2005). Briefly, 6–8 week-old, female, Swiss Webster mice were anesthetized with an intraperitoneal injection of Nembutal (50 mg/kg). The hair on the left inner thigh of each mouse was shaved and the skin was disinfected with 70% alcohol. Mice were injected subcutaneously in the inner thigh with ~10^6^ CFU either *S. aureus* (wt or Δ*spa*) or ~10^6^ *C. striatum*, or a combination of both. At 4 days post-infection, mice were euthanized and intact abscesses were harvested, weighed and placed into 2 ml of sterile PBS. Tissues were homogenized, serially diluted and plated on BHI agar with 25 μg/ml fosfomycin for *C. striatum* enumeration or MSA for *S. aureus* enumeration, to determine bacterial CFU/abscess. Experimental protocols involving mice were examined and approved by the Texas Tech University HSC Institutional Animal Care and Use Committee.

### *S. aureus* AIP1-4 GFP Fluorescence Assays

Assays to detect AIP induction were carried out similarly to those described previously, (Kavanaugh et al., 2007) using the *agrP3-gfp* AIP inducible reporter vector pDB59 (Yarwood et al., 2004). Briefly, *S. aureus* reporter strains AH1677, AH430, AH1747 and AH1871 (representing *agr* types I–IV respectively) were grown overnight in 5 ml BHI cultures at 37°C shaking at 200 RPM. From each culture, 3 ml were passed through a 0.2 μm membrane to generate *S. aureus* CFCM containing AIP produced by post-exponential phase cultures. We then generated 1.5 ml BHI cultures, which were separately inoculated with a 1:100 dilution of each overnight culture and contained a 1:10 dilution of the cognate AIP-containing overnight CFCM to induce *agr*-dependent expression of the GFP reporter as positive controls. A separate set of tubes was prepared similarly but with the addition of a 1:10 dilution of *S. aureus* CFCM containing an AIP type known to inhibit the *agr* type of the respective reporter strain; these served as negative controls for *agr* induction. *E. coli* AIP-1-containing CFCM was used to inhibit *agr* types II and III while *S. aureus* CFCM containing AIP-2 was used to inhibit *agr* types I and IV. Finally, another set of tubes was prepared identically to the positive controls except that these contained a 1:2 dilution of *C. striatum* CFCM passed through a 3 kDa-cutoff size exclusion filter. These cultures were incubated identically to the overnight cultures for either 4 or 6 h and examined for GFP fluorescence. To quantify GFP fluorescence, 25 μl of culture was combined with 75 μl of sterile BHI in a single well of a 96-well plate with four replicates per culture. GFP activity was determined by first measuring OD at 630 nm (OD_630_) then measuring GFP emission through 485 ± 20 nm excitation and 528 ± 20 nm emission filters on a BioTek Synergy HT plate reader. Fluorescence measurements were divided by OD_630_ to normalize for changes in culture density. Strains of *agr*-I, III, and IV showed maximal induction 6 h after supernatant additions whereas *agr*-II was induced at 4 h.

### Ethics Statement

This study was carried out in strict accordance with the recommendations in the Guide for the Care and Use of Laboratory Animals of the National Institutes of Health. The protocol was approved by the Institutional Animal Care and Use Committee of Texas Tech University Health Sciences Center (Protocol Number: 09039).

## Results

### Cocultivation of *S. aureus* with *C. striatum* Affects the Expression of Genes Involved in Virulence and Colonization

We hypothesized that *S. aureus* gene expression would change in response to *C. striatum*. To test this hypothesis, we assessed gene expression using RNA sequencing (RNAseq) after growing *S. aureus* JE2 (Fey et al., 2013) (a USA300 LAC derivative) in either mono- or coculture with *C. striatum* ATCC 6940 on solid (1% agarose) CDM (Brown and Whiteley, 2007) at pH 6 without glucose at 37°C. This solid-phase, low pH medium partially approximates human skin-surface conditions. There were no significant changes in growth yield, as determined by CFU (colony forming unit) measurement, between mono- and coculture conditions for either species. **Table 1** contains select *S. aureus* genes that were differentially expressed in coculture with *C. striatum* as compared to monoculture. Overall, 469 genes were differentially expressed ≥2-fold, with statistical significance (Supplementary Table S1).

A striking result from the *S. aureus* RNAseq data was that roughly half of the differentially expressed genes in cocultivation with *C. striatum* are also members of the *S. aureus agr* regulon (Cassat et al., 2006; Queck et al., 2008). The gene whose transcript was most decreased, *psmβ1*, encodes a phenol-soluble modulin toxin, which is positively regulated by the *agr* regulon, and *psmβ1* downregulation in coculture with *C. striatum* was validated using a *lacZ* reporter construct based on its own promoter (Supplementary Figure S1). Further, expression of the entire *S. aureus agr* operon was decreased in coculture with *C. striatum* (**Table 1**). Similarly, the gene whose transcript was most increased in coculture (up 260-fold) is indirectly influenced by the *S. aureus agr QS* system (Schmidt et al., 2003; Novick and Geisinger, 2008). This is the staphylococcal protein A (*spa*) gene, which encodes surface protein A (SpA). We validated this increase in *spa* transcription by qRT-PCR (**Table 1**). SpA is characterized as an immunoprotective protein that inhibits opsonization and phagocytosis (Forsgren and Sjoquist, 1966; Peterson et al., 1977; Spika et al., 1981) and recent research indicates a role for SpA during nasal colonization. Transcription of *spa* is elevated in both humans and rodents during nasal colonization compared to *in vitro* culture (Burian et al., 2010a,b; Krismer et al., 2014). In addition, during experimental *in vivo* human nasal colonization, *spa*-deficient mutants are cleared more rapidly than wild-type *S. aureus* in hosts with a robust nasal immune response and SpA protein levels positively correlate with duration of colonization (Cole et al., 2016). Interestingly, in addition to *spa, C. striatum* also induced several other *S. aureus* genes whose expression is increased during *in vivo* nasal colonization of humans (and of cotton rats), including *metI, sbnC, clfB, isdA*, and *oatA* (**Table 1**) (Burian et al., 2010a,b; Krismer et al., 2014). Overall, we observed altered levels of *S. aureus* transcripts regulated by *agr* QS during cocultivation with *C. striatum* with increased expression of genes known to be upregulated during *in vivo* nasal colonization and decreased expression of genes known to be upregulated during invasive infection. Based on these data, we hypothesized that, in response to the commensal *C. striatum*, *S. aureus* shifts to a commensal state with a decrease in activities positively regulated by the *agr QS* system and an increase in activities negatively regulated by the *agr* QS, i.e., diminished expression of virulence factors needed for successful invasive infection and increased expression of surface-associated adhesion factors important for host colonization.

### Exposure to *C. striatum* Cell-Free Conditioned Medium Is Sufficient to Alter *S. aureus agr*-Dependent Gene Expression

**FIGURE 1:**
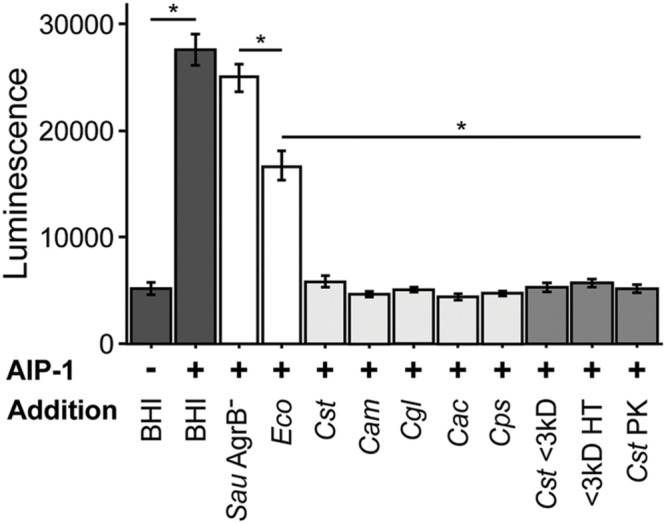
***Staphylococcus aureus agr* quorum sensing decreases in response to *Corynebacterium* spp.** Using a luminescent *agr*-I activity assay, we tested the ability of CFCM from the indicated species to inhibit activation of *agrP3* after exposure to exogenous AIP-1. Dark gray bars indicate AIP-1 negative and positive controls. White bars indicate CFCM controls from *S. aureus* JE2 containing a *Tn* insertion in *agrB* and the plasmid-free derivative of the *E. coli* strain used to exogenously produce AIP-1. Light gray bars indicate CFCM from 48 h cultures of *C. striatum*, *C. amycolatum*, *C. glutamicum*, *C. accolens*, and *C. pseudodiphtheriticum*, respectively. Medium gray bars indicate *C. striatum* (*Cst*) CFCM treatments, ‘<3 kD’ indicates sub 3 kilodalton fractions, ‘HT’ is CFCM heat treated at 95°C for 15 m, ‘PK’ indicates Proteinase K digested CFCM. For each bar, *n* = 3 and error bars represent SEM. For each result between the indicated samples (horizontal line), *p < 0.05 by two-tailed Student’s *t*-test with Bonferroni correction for multiple testing.

As the first step toward testing the hypotheses above, we asked whether the decrease in expression of genes positively regulated by *agr* QS in coculture with *C. striatum* requires cell-cell contact or whether exposure to *C. striatum* CFCM is sufficient. To address this question, we used a luminescent *agrP3* promoter reporter assay (Jensen et al., 2008), which produces light only when *agr QS* is activated, and measured the effect of *C. striatum* CFCM on *agr QS* (**Figure 1**). This also allowed us to confirm that the *S. aureus* response is mediated via *agr* QS. In this assay, *S. aureus* autoinducing peptide type 1 (AIP-1) that has been heterologously produced in *E. coli* (Thoendel and Horswill, 2009) induces an AIP-1-responsive *agrP3*-*luxCDABE* transcriptional fusion in a *S. aureus* mutant unable to produce AIP-1 (Jensen et al., 2008). If *C. striatum* CFCM is sufficient to decrease *agr* QS then addition of CFCM will decrease luminescence. *C. striatum* CFCM samples were adjusted to neutral pH to avoid inhibition of *agr* activity by pH extremes (Regassa et al., 1992). CFCM from *C. striatum* stationary phase cultures (>36 h old) was sufficient to inhibit *S. aureus agr QS* (**Figure 1**, leftmost light gray bar), indicating that cell–cell contact is not required for the observed interaction between *S. aureus* and *C. striatum*. In contrast, neither CFCM from the parent *E. coli* strain used to produce AIP-1 nor a *S. aureus* mutant with a transposon insertion in *agrB* (AgrB^-^), which is unable to produce AIP, resulted in inhibition of *agrP3*-*lux* activity to the extent of *C. striatum* CFCM (**Figure 1**, white bars). Further fractionation of *C. striatum* CFCM revealed that the inhibitory activity passed through a 3 kDa-molecular weight filter and also remained functional after heat treatment at 95°C for 15 min and was resistant to Proteinase K digestion (**Figure 1**, medium gray bars). Maximal *agr QS* inhibitory activity required preincubation of AIP-1 with *C. striatum* CFCM for at least 2 h prior to addition to the reporter strain (Supplementary Figure S2). This eliminates the possibility that inhibition is due to production of a competitive inhibitor of the AgrC sensor kinase by *C. striatum*, because an inhibitor that binds directly to AgrC would not require preincubation with AIP-1 for activity. These data established that *S. aureus* decreases activation of the *agr* QS system in response to *C. striatum* CFCM likely due to the presence of a small molecule that acts on AIP-1 itself. In subsequent experiments, we assayed for changes in *S. aureus’* activity (behavior) after exposure to *C. striatum* by using CFCM, rather than cocultivation.

### *S. aureus* Exhibits Decreased Activation of *agr QS* in Response to Multiple Commensal Species of *Corynebacterium*

In addition to *C. striatum*, *S. aureus* encounters a number of other commensal species of *Corynebacterium* in the nasal passages, on skin and in DFIs, e.g., (Citron et al., 2007; Frank et al., 2010; Wos-Oxley et al., 2010; Oh et al., 2012; Kaspar et al., 2016). To determine whether *S. aureus* responds similarly to other commensal *Corynebacterium* spp., we selected *C. amycolatum*, *C. accolens* and *C. pseudodiphtheriticum*, all three of which are common members of nasal microbiota (Yan et al., 2013; Kaspar et al., 2016), as is *S. aureus*. Also, like *C. striatum*, *C. amycolatum* is associated with DFIs (Citron et al., 2007). In addition, we tested a strain of *C. glutamicum*, which was originally isolated from soil (Abe et al., 1967) and is not associated with human colonization. When exposed to CFCM from these *Corynebacterium* spp., *S. aureus* again displayed reduced *agrP3*-*lux* reporter activity (**Figure 1**, light gray bars). These results are consistent with a conserved genus-level interaction between *Corynebacterium* spp. and *S. aureus* that is predicted to result in a shift in *S. aureus* behavior toward colonization and away from virulence. We selected *C. striatum* for all subsequent experiments to characterize the *S. aureus* phenotypic response to commensal *Corynebacterium* spp.

### *S. aureus* Adhesion to Epithelial Cells Increases When Exposed to *C. striatum*

**FIGURE 2:**
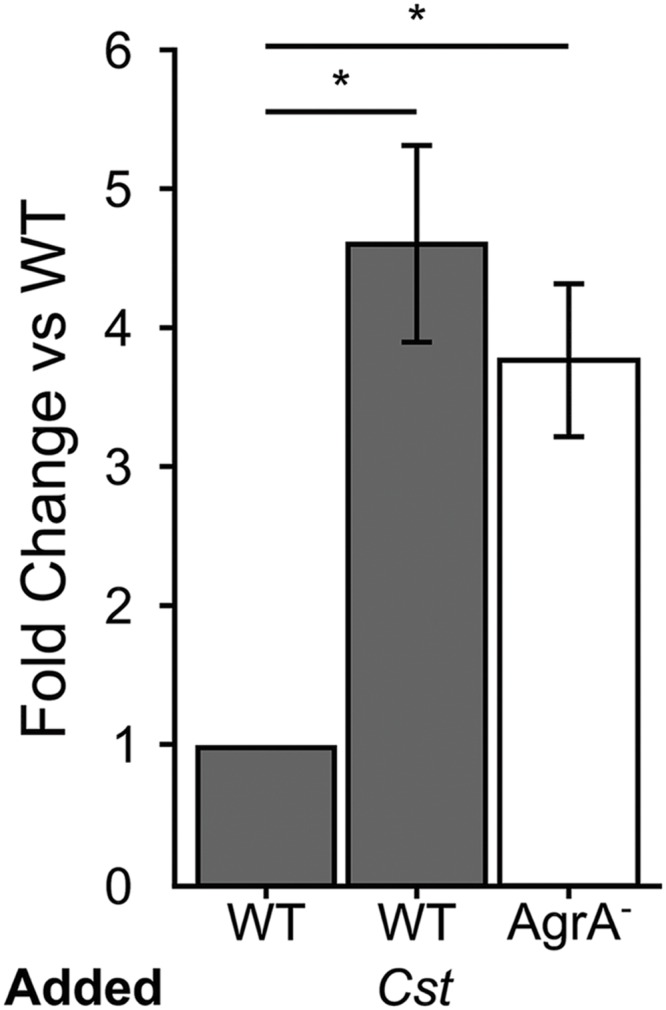
***Staphylococcus aureus* attachment to human epithelial cells increases in response to *C. striatum* CFCM.** When *S. aureus* WT (gray bars) and AgrA^-^ (white bar) cells were exposed to AIP-1 plus or minus *C. striatum* CFCM (*Cst*), we observed 4.5- and 3.6-fold increases in attachment respectively. These increases were statistically significant (*) when compared to the WT alone. *S. aureus* attachment to human A549 epithelial cells was quantified after 1 h of exposure. Attachment was measured as the percentage of attached cells divided by the total number of *S. aureus* planktonic cells added, as determined by CFU enumeration. Fold change was determined by dividing the percent attached for each condition by that of the % attached for WT exposed only to AIP. Error bars were omitted from the WT normalized data for clarity. Data were analyzed by two-tailed Student’s *t*-test with Bonferroni correction for multiple testing (*p < 0.03). Error bars represent SEM.

Many of the cell surface proteins that are expressed when the *S. aureus agr* QS is not activated are involved in adhesion to host cells (Burian et al., 2010b; Foster et al., 2014), and are negatively regulated by activation of *agr* QS. Therefore, we hypothesized that when grown to high cell density in the presence of *C. striatum* CFCM, *S. aureus* would exhibit increased adhesion to host epithelial cells, a phenotype that can serve as a proxy for colonization or commensal behavior (Foster et al., 2014). To test this, we measured *S. aureus* attachment to A549 human airway epithelial cells, similarly to previously described (Weidenmaier et al., 2004). We cultured wild-type *S. aureus* in the presence of exogenous AIP-1 plus or minus *C. striatum* CFCM. As a positive control, we performed the identical experiment with an isogenic *S. aureus agrA*-deficient mutant in the presence of AIP-1. *S. aureus* cells in late exponential phase from each condition were normalized to identical optical densities and then added to epithelial cell monolayers at a multiplicity of infection (MOI) of 25. Next, we enumerated total planktonic and epithelial-cell-attached *S. aureus* by CFU measurement. We compared the proportion of planktonic versus epithelial cell-attached CFUs in each condition and expressed the results as fold change of attached cells from each condition compared to the *S. aureus* WT. After growth with *C. striatum* CFCM, the wild-type *S. aureus* (WT; dark gray bars in **Figure 2**) exhibited increased adhesion to the respiratory epithelial cells; this increase was comparable to that of an isogenic *agrA*-deficient mutant (AgrA^-^, white bar in **Figure 2**). The increase in *S. aureus* adhesion to epithelial cells when exposed to *C. striatum* is consistent with our hypothesis that *S. aureus* shifts toward a commensal state in the presence of *C. striatum*.

### *S. aureus* Increases SpA on Its Surface in Response to *C. striatum*

**FIGURE 3:**
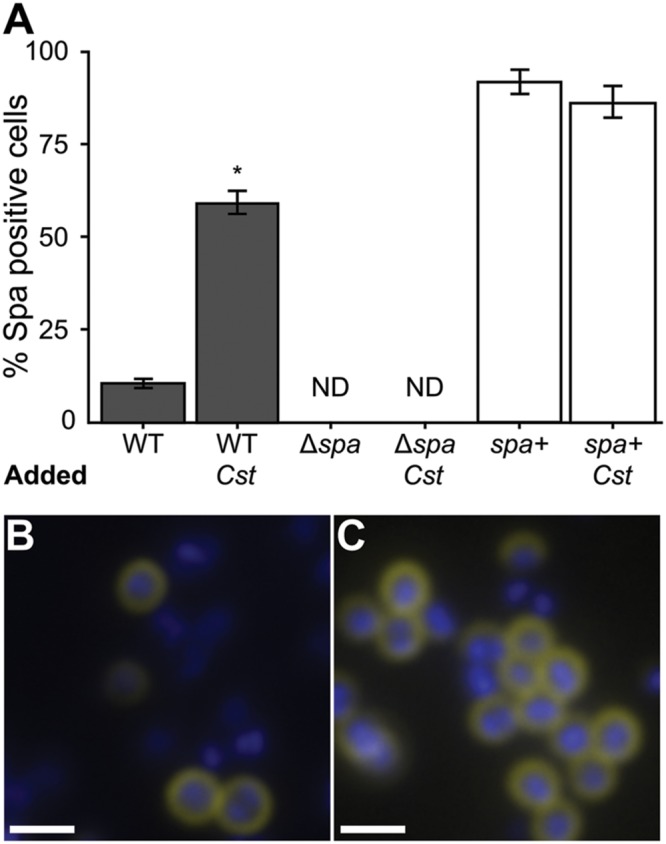
**The proportion of *S. aureus* cells with active surface-associated SpA increases in coculture with *C. striatum*.** *S. aureus* strains were grown in mono- versus coculture with *C. striatum* under conditions identical to those used in **Table 1**. SpA activity was detected by observing SpA-mediated capture of FITC-conjugated goat IgG (yellow in **B,C**). Cells were counterstained with DAPI (blue in **B,C**). Cell intensity was quantified for all *S. aureus* strains in three fields of view at 1000x magnification each from 3 biological replicates under identical exposure settings. **(A)** ImageJ software was used to count total cells versus FITC-positive cells. 10% of wild-type *S. aureus* (WT) were positive for SpA activity in monoculture compared to 60% in coculture with *C. striatum* (*Cst*). The *spa*-deletion mutant carrying the empty expression vector (Δ*spa*) had no detectable FITC staining (ND). The *spa*-deletion mutant carrying an expression vector expressing *spa* from its native promoter (*spa*+) had FITC-positive cells in both conditions consistent with this construct producing SpA above wt levels. Error bars represent SEM. Data were analyzed by two-tailed Student’s *t*-test with Bonferroni correction for multiple testing (*p < 0.003). Representative micrographs of wild-type *S. aureus* cells (blue) in mono- **(B)** versus coculture **(C)** stained for SpA (yellow) at the cell surface. Scale bars represent 1.5 μm.

The *S. aureus spa* gene transcript was the most highly increased in coculture with *C. striatum* compared to monoculture. Traditionally, SpA has been studied in the context of invasive infection; however, using qRT-PCR, Burian et al. (2010b) report that transcript levels of *spa* are increased *in vivo* during nasal colonization compared to *in vitro* culture indicating SpA might play a role in commensal interactions with the host. More recently, Cole et al. (2016) found that increased levels of SpA correlate with longer duration of *S. aureus* colonization during experimental human nasal inoculation and that *spa*-deficient mutants are less effective than the wild-type at colonizing some humans. Based on these data, which indicate a likely role for SpA in colonization, we proceeded to explore the effect of the observed increase in *spa* transcription in response *C. striatum.* Regulation of *spa* transcript levels is well studied and complex, depending on several elements (Schmidt et al., 2003; Gustafsson et al., 2009). Like many adhesion factors, SpA is a cell-surface protein that is negatively regulated through *agr* QS, albeit indirectly. Therefore, we first verified that the increase in *spa* transcription during cocultivation with *C. striatum* was indeed dependent on *agrA* (Supplementary Figure S3). We hypothesized that the increase in *spa* transcript level when grown with *C. striatum* would translate to an increase in *S. aureus* production of functional SpA. To test this hypothesis, we investigated SpA abundance and activity in *S. aureus* using immunoblot and immunofluorescence assays. First, we stained for SpA via immunoblot with an α-SpA antibody using cell lysates from cultures incubated in the presence of AIP-1 alone or with exposure to *C. striatum* CFCM. SpA abundance qualitatively increased upon treatment with *C. striatum* CFCM and SpA was absent in a Δ*spa* mutant (Supplementary Figure S4). We then assayed for SpA activity with a standard IgG binding assay. Using solid-phase culture conditions identical to those in our RNAseq experiments, we quantified the proportion of *S. aureus* cells exhibiting SpA IgG binding activity in mono- versus coculture by measuring SpA capture of FITC-conjugated IgG antibodies. We found that 60% of wt *S. aureus* cells had detectable IgG capture in coculture compared to only 10% in monoculture (**Figure 3A**, dark bars). IgG capture was visible as a ring around cells in both mono- and coculture with *C. striatum*, consistent with cell-surface expression of SpA (**Figures 3B,C**). No FITC fluorescence was detected in a Δ*spa* mutant in mono- or coculture (**Figure 3A**). Fluorescence signal was restored when *spa* was complemented *in trans*. The greater proportion of cells with detected surface IgG capture in the Δ*spa* mutant containing a multicopy plasmid with *spa* under its native promoter suggests that the construct results in expression of *spa* above wt levels (**Figure 3A**, white bars). Together these data demonstrate that the *C. striatum*-dependent increase in *S. aureus spa* transcript levels (**Table 1**) results in a phenotypic change with an increased number of cells displaying active SpA on their surface. Active SpA can diminish opsonization and phagocytosis via binding of the Fc portion of IgG, inhibiting serum complement pathway activation (Forsgren and Sjoquist, 1966; Peterson et al., 1977; Spika et al., 1981). Therefore, we also confirmed that the increased level of SpA activity in coculture corresponded to decreased *S. aureus* phagocytosis (Supplementary Figure S5). Together with the evidence for increased epithelial cell adhesion after exposure to *C. striatum*, these results, along with reports that *spa* expression and SpA abundance increases during *in vivo* nasal colonization (Burian et al., 2010b; Cole et al., 2016), support our hypothesis that *S. aureus* shifts toward a colonization (commensal) state in the presence of *Corynebacterium* spp.

### *S. aureus agr*-Dependent Hemolytic Activity Decreases in Response to *C. striatum*

**FIGURE 4:**
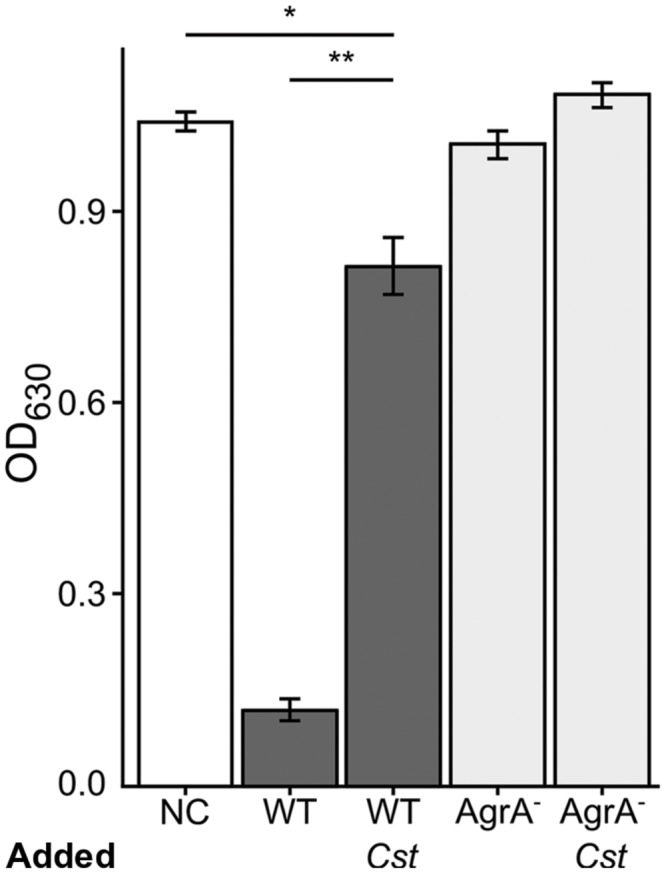
***Staphylococcus aureus* exhibits decreased hemolytic activity when grown with *C. striatum* CFCM.** Hemolysis of rabbit erythrocytes was quantified 30 m after exposure to BHI, as the negative control (NC), or CFCM from *S. aureus* strains grown in the presence of AIP-1 alone or plus the addition of *C. striatum* CFCM (*Cst*) for the wild-type (WT) and *agrA::Tn* mutant (AgrA^-^), *n* = 3 each. Decreased OD_630_ is indicative of *S. aureus* hemolytic activity. Growing *S. aureus* in the presence of *C. striatum* CFCM significantly diminished the hemolytic activity produced by the WT. In contrast, the *agrA::Tn* mutant (AgrA^-^) was incapable of significant hemolysis in either condition. Data were analyzed by two-tailed Student’s *t*-test with Bonferroni correction for multiple testing (*p < 0.005, **p < 0.00005). Error bars represent SEM.

The other side of *agr* QS-regulated activities in *S. aureus* is an increase in the production of secreted virulence factors at high cell density when *agr* QS is activated (Novick and Geisinger, 2008; Thoendel et al., 2011). Hemolysis has traditionally served as an approximation of *S. aureus* virulence factor production and is typically diminished in *agr* mutants (Blevins et al., 2002). Therefore, we assayed for the rabbit erythrocyte hemolytic activity of CFCM from *S. aureus* grown with or without *C. striatum* CFCM. Based on our RNAseq data, we predicted that *S. aureus* exposed to *C. striatum* would exhibit decreased hemolysin activity due to repression of genes encoding β and δ-hemolysins (**Table 1**), which are both positively regulated by *agr* QS. Using a published method (Pang et al., 2010), we quantified hemolysis as the loss of optical density at 630 nm of rabbit erythrocytes when mixed with CFCM harvested from each *S. aureus* strain induced with AIP-1, in late-exponential phase, in the presence or absence of *C. striatum* CFCM (*Cst*). An *agr*A-deficient mutant served as a non-hemolytic control (**Figure 4**, light gray bars). In this assay, wild-type *S. aureus* production of hemolytic activity was strongly diminished by exposure to C. *striatum* CFCM (**Figure 4**, dark gray bars). These results further support our hypothesis that *S. aureus* shifts away from virulence in the presence of *C. striatum*.

### *S. aureus* Numbers Decrease during Coinfection with *C. striatum* in a Murine Abscess Model

**FIGURE 5:**
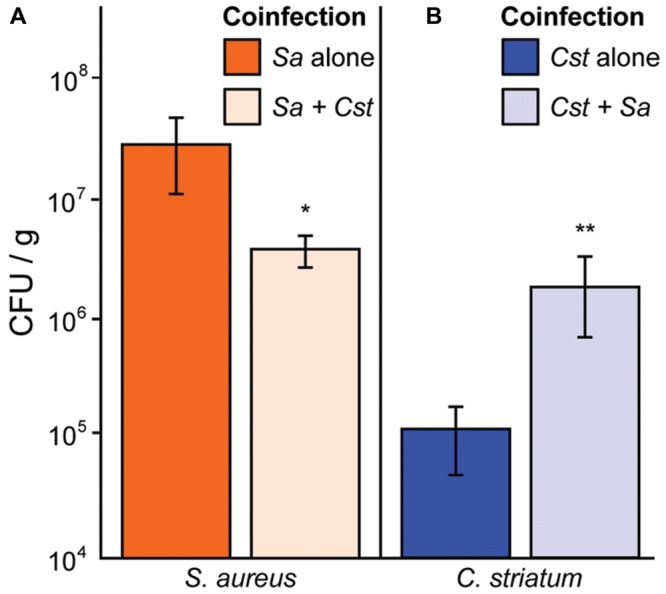
***Staphylococcus aureus* abundance decreases *in vivo* when coinfected with *C. striatum* in a murine abscess model. (A)** In a murine abscess infection model 4 days post-infection, wild-type *S. aureus* showed reduced numbers (CFU/g) during coinfection with *C. striatum* (light orange bar; *Sa* + *Cst*) compared to monoinfection (orange bar; *Sa* alone). **(B)** In the same model, *C. striatum* numbers increased significantly when coinfected with *S*. *aureus* (light blue bar; *Cst* + *Sa*) when compared to monoinfection (blue bar; *Cst* alone). For each bar, *n* = 9. Data were analyzed using the Mann Whitney *U*-test (*p < 0.03, **p < 0.02). Error bars represent SEM.

We have shown that *S. aureus agr* QS-dependent gene expression is altered in response to commensal *Corynebacterium* spp. in a manner that shifts its phenotypic behavior toward colonization and away from virulence. Therefore, we hypothesized that *in vivo* coinfection with *C. striatum* would attenuate *S. aureus’* success when compared to monoinfection. To test this, we compared colony-forming units (CFUs) from mono- and coinfection of *S. aureus* and *C. striatum* in a previously established murine subcutaneous abscess model (Mastropaolo et al., 2005; Ramsey et al., 2011). We measured abscess weight and bacterial CFUs 4 days after subcutaneous injection of single- versus dual-species infections. *S. aureus* CFUs decreased 6.2-fold in coinfection with *C. striatum* (**Figure 5A**), whereas *C. striatum* CFUs increased 20.7-fold in coinfection with *S. aureus* (**Figure 5B**). Thus, coinfection with *S. aureus* and *C. striatum* resulted in a disadvantage for *S. aureus* while providing an advantage to *C. striatum* compared to monoinfection, implying that *S. aureus* responds to the presence of *C. striatum* with changes in gene/protein expression that decrease its fitness for invasive infection while *C. striatum* capitalizes on the presence of *S. aureus* in this infection model.

### *S. aureus* AIP Types 1, 2, and 3 Respond Similarly to *C. striatum*

**FIGURE 6:**
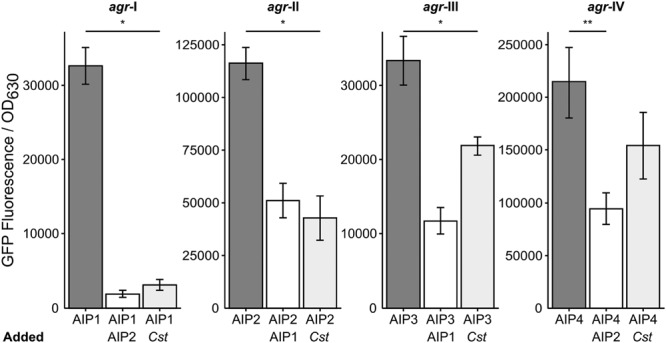
***Corynebacterium striatum* CFCM inhibits signaling of *S. aureus agr* types I, II, and III.** For each respective *agr* type, post-exponential phase *S. aureus* CFCM from that same type was used to induce an *agrP3:gfp* reporter. CFCM containing AIP-1 was used to inhibit *S. aureus agr* types II and III strains and CFCM containing AIP-2 was used to inhibit *S. aureus agr* types I and IV strains (white bars). Cultures grown in the presence of inducing AIP-containing *S. aureus* CFCM and *C. striatum* CFCM showed pronounced inhibition of *agr* types I, II, and III and no significant inhibition of *agr* type IV (light gray bars). GFP was measured as fluorescence/OD_630_ (*n* = 3). Error bars represent SEM. Data were analyzed by two-tailed Student’s *t*-test with Bonferroni correction for multiple testing (*p < 0.005 and **p < 0.01).

*Staphylococcus aureus* strain JE-2, which was used for all of the above experiments, is an AIP type I strain. *S. aureus* has four characterized *agr* types, (*agr* I–IV) that each make a specific AIP molecule (AIP 1–4) along with its cognate AgrC (reviewed in Novick and Geisinger, 2008; Thoendel et al., 2011). Each AIP type acts as a potent quorum signal inhibitor for other *agr* type strains, with the exception of AIP-1 and AIP-4 whose structures differ by only a single peptide (D-Y substitution) and which do not inhibit each other (Novick and Geisinger, 2008; Thoendel et al., 2011). We hypothesized that multiple *agr* types would respond similarly to *C. striatum* and tested this using a published assay (Kavanaugh et al., 2007) with an AIP-inducible *agrP3-gfp* transcriptional fusion to monitor *agr* QS-dependent activation in strains representing all four *agr* types. In liquid culture, the *agr* QS system is activated in stationary phase when AIP levels are highest; therefore, as a positive control, we added stationary-phase CFCM from each cognate AIP-type strain as an inducer to mid-exponential-phase cells of the same strain and measured GFP fluorescence (**Figure 6**, dark gray bars). Strains incubated with a combination of the cognate *S. aureus* AIP CFCM and an inhibitory *S. aureus* AIP-type CFCM served as negative controls (**Figure 6**, white bars). Reporter cultures were incubated with a mix of cognate inducing *S. aureus* AIP CFCM and *C. striatum* CFCM to test for inhibition. In response to *C. striatum* CFCM, the *agrP3* activity of *agr* types I, II, and III decreased, but type IV’s did not (**Figure 6**, light gray bars). These results indicate that in response to *C. striatum*, and likely *Corynebacterium* spp. in general, *agr* QS decreases in multiple *S. aureus agr* lineages.

## Discussion

*Staphylococcus aureus* is a common cause of infection in humans, and nasal colonization correlates with an increased risk of infection (Wertheim et al., 2005). *S. aureus* infections range from chronic and indolent, e.g., polymicrobial DFIs, to acute and aggressive, e.g., monomicrobial bacteremia. However, *S. aureus* is typically an asymptomatic colonizer of humans (Kuehnert et al., 2006; Gorwitz et al., 2008), coexisting in the nasal and skin microbiota with other bacteria, including members of the genus *Corynebacterium*, e.g., (Uehara et al., 2000; Lina et al., 2003; Wos-Oxley et al., 2010; Oh et al., 2012; Yan et al., 2013). Therefore, from both a clinical and public health perspective, it is important to identify factors that influence whether *S. aureus* behaves as a commensal or a pathogen. This is highlighted by the fact that *S. aureus* has eluded repeated attempts at vaccine development (Proctor, 2012; Jansen et al., 2013), accentuating the need for alternative approaches to prevent *S. aureus* infections. *Corynebacterium* spp. commonly coexist with *S. aureus* on epithelial surfaces of the nasal passages and skin, as well as in DFIs (Uehara et al., 2000; Lina et al., 2003; Citron et al., 2007; Frank et al., 2010; Wos-Oxley et al., 2010; Oh et al., 2012; Gardner et al., 2013; Yan et al., 2013); yet, little is known about how *S. aureus* interacts with *Corynebacterium* spp. Here, we have demonstrated that *S. aureus* responds to commensal *Corynebacterium* spp. with altered expression of genes involved in colonization and virulence (**Table 1**, Supplementary Table S1), and does so in a manner that is similar to the transcriptomes of *agr* QS loss-of-function mutants (Dunman et al., 2001; Cassat et al., 2006; Queck et al., 2008). This transcriptional response translates to an increase in cell-surface activities associated with colonization, e.g., epithelial-cell adhesion and SpA activity (**Figures 2** and **3**), and a decrease in production of secreted virulence factors, e.g., hemolysin (**Figure 4**). During *in vivo* infection, these result in decreased success during coinfection with *C. striatum*. In total, the data presented here indicate that *S. aureus* responds to commensal *Corynebacterium* with a shift to commensalism. This opens up the possibility that commensal *Corynebacterium* spp. are an unexplored source for new antivirulence therapies that limit activation of *S. aureus agr* QS as a means to control and/or prevent *S. aureus* infection, a goal that has been actively explored through other approaches (Murray et al., 2014; Nielsen et al., 2014; Sully et al., 2014).

**FIGURE 7:**
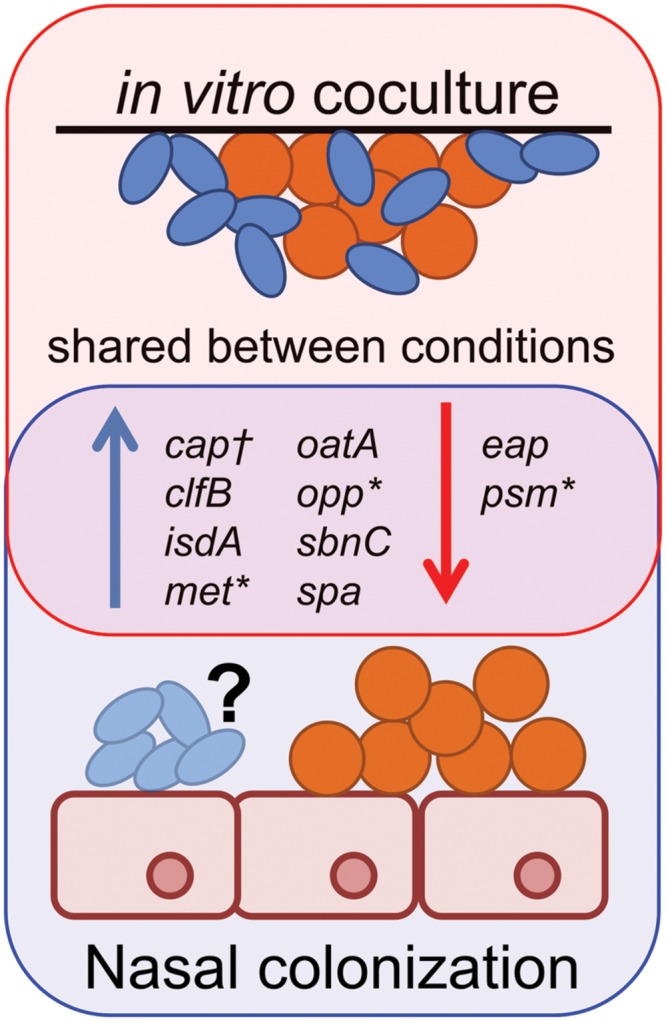
***Staphylococcus aureus* gene expression during *in vivo* colonization is similar to *in vitro* coculture with *C. striatum*.** Gene expression data from *S. aureus* (orange spheres) *in vivo* colonization of humans (Burian et al., 2010b; Krismer et al., 2014), cotton rats (Burian et al., 2010a) or *in vitro* coculture with *C. striatum* (blue ovals) (**Table 1**/Supplementary Table S1) is depicted as upregulated (blue arrows) or downregulated (red arrows) in comparison to *in vitro* monoculture. Genes quantified by qRT-PCR *in vivo* share similar patterns of expression with those detected with RNAseq during *in vitro* coculture with *C. striatum* (“shared between conditions”). *In several cases, individual genes identified by qRT-PCR were part of operons whose members were also differentially regulated in our RNASeq results (Supplementary Table S1). †Type 5 capsule (CP5) production was quantified by ELISA in a mouse nasal colonization model (Kiser et al., 1999) and was overrepresented *in vivo* versus *in vitro*. We observed upregulation of several CP5 synthesis genes in coculture with *C. striatum* (Supplementary Table S1). It is unknown (“?”) whether or not *Corynebacterium* spp. were present in the referenced *in vivo* experiments. These data demonstrate the similarities in *S. aureus* gene expression during host commensal *in vivo* colonization and *in vitro* growth with *Corynebacterium* spp.

Our *in vitro* coculture RNAseq data showed striking similarities to *S. aureus* gene expression during *in vivo* nasal colonization of humans, and rodent models of *S. aureus* nasal colonization (Kiser et al., 1999; Burian et al., 2010a,b; Krismer et al., 2014) (**Figure 7**). For example, Krismer et al. (2014) suggest that methionine competition and synthesis, along with oligopeptide transport and iron acquisition, might be critical for *S. aureus* colonization, based on finding that *metI*, which encodes a methionine biosynthesis gene; *oppB*, which encodes an oligopeptide transporter; and *sbnC*, which encodes an iron transport protein, are induced during human nostril colonization. Indeed, a *metI*-deficient mutant has strongly reduced colonization capacity in the cotton rat model of *S. aureus* nasal colonization (Krismer et al., 2014). Our transcriptome data show that the levels of *metI*, and the methionine synthesis operon it is part of (SAUSA300_0357-360; **Table 1**/Supplementary Table S1), are increased in the presence of *C. striatum* suggesting that *S. aureus* and *Corynebacterium* may compete for methionine *in vitro* in methionine-replete medium, as well as *in vivo*. We did not detect significant differential expression of *oppB*; however; we did observe upregulation of six other *opp* genes (Supplementary Table S1), two of which are adjacent to *oppB*, suggesting *S. aureus* may respond similarly during coculture with *C. striatum* and *in vivo* nasal colonization. We also found that *sbnC* and its operon (SAUSA300_0118-122; **Table 1**/Supplementary Table S1) were upregulated in coculture with *C. striatum* along with iron-regulated surface determinant adhesin-encoding *isdA* (**Table 1**/Supplementary Table S1) indicating another possible competition for iron.

In another study, Burian et al. (2010b) selected target genes to reflect functions that distinguish colonization from invasive infections, e.g., adhesins versus secreted toxins (Burian et al., 2010b), and then used qRT-PCR to examine transcription of these 30 *S. aureus* genes during human nostril colonization. They report increased transcript levels of *spa*, the clumping factor B adhesin-encoding *clfB* [which is reported to be a major determinant in *S. aureus* human nasal colonization (Wertheim et al., 2008)], *isdA* and the secretory antigen *oatA* in human nostrils compared to *in vitro* culture, along with decreased expression of the *psmβ1-2* genes (Burian et al., 2010b). Again, these changes in *S. aureus* transcription are similar to our *in vitro* coculture data (**Table 1**; Supplementary Table S1, **Figure 1**; Supplementary Figure S1). However, unlike their *in vivo* colonization data, we did not observe differential expression of *sceD*, *atlA*, *sak, hla* and wall teichoic acid related genes (*tagO* and *tarK*) (Supplementary Table S1), which suggests that the increase in these might be a response to the host environment or to other commensal bacteria.

*Staphylococcus aureus* nasal colonization has also been studied in rodent models. (Of note, at present, there are no established models for *Corynebacterium* spp. nasal colonization.) During stable late-term *S. aureus* nasal colonization of the cotton rat, another study found increased expression of *clfB* and *isdA* (Burian et al., 2010a), both of which were upregulated in coculture with *C. striatum in vitro* (**Table 1**/Supplementary Table S1). Additionally, Kiser et al. (1999) observed that *S. aureus* type-5 capsule (CP5) is produced in greater abundance in a mouse nasal colonization model than *in vitro* and that CP5 is necessary for abundant nasal colonization. Consistent with this, we detected five *S. aureus* CP5 biosynthesis genes upregulated in coculture with *C. striatum* (Supplementary Table S1).

There are two caveats in comparing our *in vitro* transcription data to data from *in vivo* nasal colonization. First, we do not know the composition of the nasal microbiota in the human or rodent colonization studies. Second, our conditions do not perfectly mimic the host environment. However, our data demonstrate that many of the key genes expressed during *S. aureus* nasal colonization of humans and rodents are also expressed in the presence of *C. striatum* in the complete absence of a mammalian host. This suggests the *S. aureus* response to *Corynebacterium* spp. in the microbiota contributes to reported *in vivo* expression changes. This type of observation is not unprecedented; Ramsey and Whiteley (2009) have previously shown in an unrelated study that a commensal bacterium alone induces an immunoprotective response in an opportunistic pathogen *in vitro*. In fact, it is possible that many of the transcriptional responses observed in the microbiota may be driven by microbe–microbe interactions and not simply by host–microbe interactions.

Based on results presented here, we propose that the *S. aureus* response to commensal *Corynebacterium* in polymicrobial infections dampens its virulence with implications for its behavior during both colonization and polymicrobial infection. *S. aureus* is capable of causing acute, destructive monomicrobial infections through the production of secreted virulence factors regulated by *agr QS* and other pathways (Lowy, 1998; Otto, 2010). However, in some chronic infections, *S. aureus* displays a loss of *agr* function, e.g., persistent bacteremia (Fowler et al., 2004) and infections of the cystic fibrosis lung (Goerke et al., 2000). Another type of chronic infection is the DFI, where *S. aureus* is a prominent member and positively correlates with the presence of *Corynebacterium* spp. (Gardner et al., 2013). We observed that *S. aureus agr* QS is mitigated in response to multiple *Corynebacterium* spp. across several *agr* classes (**Figures 1** and **6**) and that this results in a lack of hemolytic activity, which represents a lack of production of secreted virulence factors (**Figure 4**) and decreased success during *in vivo* coinfection in a mouse subcutaneous abscess model (**Figure 5**). Thus, in response to phylogenetically diverse *Corynebacterium* spp., *S. aureus* shifts toward a commensal (i.e., less virulent) state reminiscent of *agr*-defective mutants. We speculate that this contributes to a shift from acute to chronic *S. aureus* infection in polymicrobial settings and, along with an increase in the abundance of *C. striatum* in coinfection (**Figure 5**), may partly explain the positive correlation between *S. aureus* and *Corynebacterium* spp. in chronic DFIs.

Although the mechanism remains to be determined, *S. aureus’* response to *C. striatum* is reminiscent of how *Lactobacillus reuteri*-produced cyclic dipeptides inhibit *S. aureus agr* QS and diminish TSS-1 production (Li et al., 2011). A number of distinct mechanisms could result in a similar diminution of *agr* QS and we are actively pursuing the identity and mechanism of the activity in *C. striatum* CFCM that triggers this response.

Overall, our results point to the potential to develop antivirulence therapies against *S. aureus* from *Corynebacterium*-produced products and also suggest a potential reason for the high frequency of commensal behavior by *S. aureus* during human nasal colonization. Research on nostril microbiota composition and observed correlations, both positive and negative, between the presence/relative abundance of *S. aureus* and commensal *Corynebacterium* spp., e.g., (Uehara et al., 2000; Wos-Oxley et al., 2010; Yan et al., 2013; Kaspar et al., 2016), have sparked renewed interest in the potential use of commensal *Corynebacterium* spp. as probiotics to eradicate *S. aureus* nostril colonization, and there is precedent for this in a small cohort of adults (Uehara et al., 2000). Our findings suggest the possibility of an alternative or additional role of probiotic *Corynebacterium* spp. in limiting *S. aureus* virulence, e.g., in persistent carriers. In addition, our results provide an additional impetus for the development of an animal model of *Corynebacterium* spp. nasal colonization. Future efforts to fully characterize and manage *Corynebacterium–S. aureus* interactions have the potential to either maintain healthy microbiota composition or attenuate local *S. aureus* infections and may lead to new minimally invasive therapeutic adjuncts and/or alternatives to antibiotic treatment.

## Author Contributions

Conceptualization, MR and KL; Methodology, MR, KL, KR, and MF; Investigation, MR, MF, and RG; Writing – Original Draft, MR and KL; Writing – Review and Editing, MR., KL, KR; Funding Acquisition, KL and KR; Supervision, KL and KR. All authors agree to be accountable for the content of the work.

## Conflict of Interest Statement

The authors declare that the research was conducted in the absence of any commercial or financial relationships that could be construed as a potential conflict of interest.
