# Supplementary material for: Staphylococcus aureus Shifts toward Commensalism in Response to Corynebacterium Species
Source: Front Microbiol. 2016 Aug 17;7:1230. doi: 10.3389/fmicb.2016.01230 (PMC4988121; doi:10.3389/fmicb.2016.01230)
Supplement: Supplementary file 1 [file Data_Sheet_1.DOCX]

**Supplemental Materials and Methods**

**Construction of *lacZ* transcriptional reporter fusions.** Construction of the *lacZ* promoterless transcriptional reporter vector pMR370 was performed by modification of the constitutive *lacZ* expression vector pAJ22 (O'Neill et al., 2004) by replacing the *lacZ* gene and constitutive promoter. To reinsert a promoterless *lacZ* gene including an upstream BamHI site, a 3.5 kb fragment amplified from pAJ22 with primers oKL305 and oKL304 was digested with NotI and SalI and then ligated with the gel-purified NotI-SalI digested 5.8 kb vector-only fragment of pAJ22 to yield pMR370. Then pMR370 was digested with BamHI and SalI and ligated with amplicons digested with identical enzymes amplified by oKL308 and oKL319 to generate the *spa* promoter (P*_spa_-lacZ*) reporter vector pMR371; and by oKL379 and oKL380 to generate the SAUSA300_1067 promoter (P*_psmβ1_-lacZ*) reporter vector pMR372.

**β-galactosidase assays.** β-galactosidase assays were performed as previously described (Steinmoen et al., 2002) with the exception of *S. aureus* lysis, which was performed by incubation in 100µL TE buffer with 0.2% Triton X-100 and 25 µg/ml lysostaphin. β-galactosidase activity was calculated in Miller units (Miller, 1972).

**Immunoblot procedure.** *S. aureus* was cultured as described in the “*S. aureus* rabbit erythrocyte lysis assay” and 5 x 10^9^ *S. aureus* cells (as estimated by an OD_600_ of 1 = 10^9^ cells) were pelleted by centrifugation at 10,000*g* for 10 m. For lysis, cell pellets were resuspended in 500 µl PBS with 25 µg/ml lysostaphin and incubated 30 m at 37°C. After incubation, 25 µl of 10% Triton X-100 was added to each sample and incubated for 10 m at 37°C. Samples were then mixed at a 1:1 volume ratio with Laemmli sample buffer (BioRad) and incubated at 95°C for 10 m. An aliquot of each sample was preserved prior to Laemmli buffer addition for protein quantification via Bradford assay. From each sample, 125 ng of protein (~15 µl) was added to each well of a 4-20% HEPES polyacrylamide gradient gel (Pierce). Gels were run at 100 mV for ~50 m until the dye front reached the bottom of the gel. Gels were placed against nitrocellulose membranes and proteins were transferred at 20 V for 1 h using a BioRad Transblot SD and 1x transfer buffer (Boston Bioproducts) per the manufacturers’ instructions. Membranes were blocked in PBS with 5% milk powder (Carnation) for 1 h at RT. Membranes were then incubated in 15 ml blocking solution containing a 1:15,000 dilution of α-SpA-HRP conjugated antibody (ThermoFisher) under gentle agitation for 1 h at RT. Membranes were washed 3x for 5 m intervals in PBS with 0.1% Tween 80 and given a final rinse in PBS. Membranes were then exposed to 200 µL LumiGlo chemiluminescent substrate (KPL Biosciences) and incubated in the dark at RT for 5 m. Excess liquid was blotted off with paper and blots were imaged for luminescence in an AlphaImager FluorchemQ (Alpha Innotech) for 5 m with no filters selected. Blot images were captured and manipulated using Adobe Photoshop. Images are from full single gels and are not composites from different gels.

**Phagocytosis assays.** Human phagocytic HL-60 cells were subcultured into RPMI 1640 medium (Gibco, USA) supplemented with 10% heat-inactivated FBS, 5.5 mM glucose, 1% DMSO (Sigma, USA) and incubated for 6 d at 37°C to differentiate cells (Ayilavarapu et al., 2010). Cells were then counted and aliquoted in 1 x 10^6^ increments. We added a 1:10 dilution of *E. coli* CFCM containing AIP-1 into 1.5 ml cultures containing 1:100 inoculums of either wild-type or *spa*-deficient *S. aureus* JE2. A separate set of tubes was prepared identically plus the addition of a 1:2 dilution of 3 kDa-filtered *C. striatum* CFCM. These cultures were incubated at 37°C shaking at 200 RPM for 4 h. Next, 2 x 10^7^ *S. aureus* cells from each growth condition (as estimated by an OD_600_ of 1 = 10^9^ cells) were labeled with BacLight Green (Molecular Probes) for 20 m at RT with gentle agitation and washed twice with PBS. Labeled bacteria were opsonized in heat inactivated normal serum (Sigma) for 30 m at RT and incubated with differentiated HL-60 cells (1:20 ratio) for 1 h in serum and antibiotic-free medium at 37°C. Cells were gently washed, extracellular fluorescence was quenched by Trypan blue and phagocytosis was determined by flow cytometry analysis (BD FACS Aria). Positive labeled cells were evaluated by Flow-Jo and data expressed as phagocytic index: Phagocytic index = % phagocytic neutrophils × mean fluorescence intensity (Drevets et al., 2015).

**S2 Table. Strains and plasmids used in this study.**

|  | Strain | Internal Reference | Details | Reference |
| --- | --- | --- | --- | --- |
| Plasmids |  |  |  |  |
| pAIP1 | AH594 | KPL2428 | P_BAD_-*agrBD* vector for inducible synthesis of *S. aureus* AIP-1 in *E. coli* ER2566 | (Thoendel and Horswill, 2009) |
| pAJ22 | KPL2468 | KPL2468 | *lacZ* overexpression vector for *S. aureus* in *E. coli* NEB5-α | (O'Neill et al., 2004) |
| pMR370 | KPL2469 | KPL2469 | promoterless *lacZ* expression vector for *S. aureus* in *E. coli* NEB5-α | this study |
| pMR371 | KPL2470 | KPL2470 | P*_spa_-lacZ* reporter in *E. coli* NEB5-α | this study |
| pMR372 | KPL2513 | KPL2513 | P*_psmβ1_-lacZ* reporter in *E. coli* NEB5-α | this study |
| pLF048 | LCF048 | KPL2512 | a pKFT-derivative Ts vector for in-frame deletion of *spa* locus in *S. aureus* RN4220 | (Foulston et al., 2014) |
| pEPSA5 | KPL2283 | KPL2283 | *E. coli*, xylose inducible *S. aureus* shuttle vector in *E. coli* NEB5-α | (Forsyth et al., 2002) |
| pEPSA5-*spa* | KPL2494 | KPL2494 | *spa* expression vector in *E. coli* NEB5-α | this study |
| Species |  |  |  |  |
| *E. coli* | NEB5-α |  | Cloning strain | neb.com |
| *C. accolens* | DSM 44278 | KPL2060 | Type strain | (Neubauer et al., 1991) |
| *C. amycolatum* | DSM 6922 | KPL2422 | Human skin isolate | (Collins et al., 1988) |
| *C. glutamicum* | DSM 20300 | KPL1958 | Soil isolate derivative | (Abe et al., 1967) |
| *C. pseudodiphtheriticum* | DSM 44287 | KPL1953 | Type strain | (Riegel et al., 1996) |
| *C. striatum* | ATCC 6940 | KPL1959 | Human skin isolate | (Pitcher, 1983) |
| *S. aureus* | RN4220 | KPL2077 | Cloning strain | (Kreiswirth et al., 1983) |
| *S. aureus* | ROJ143/ AH1229 | KPL2430 | ∆*agr*::*ermB* + pArC/*agrA agrP3-luxABDCE* (cm^R^) in RN4220 | (Jensen et al., 2008) |
| *S. aureus* | AH1677 | KPL2431 | pDB59 (cm^R^) in *S. aureus* LAC (*agr* type I *agrP3-gfp* reporter strain) | (Kirchdoerfer et al., 2011) |
| *S. aureus* | AH430 | KPL2432 | pDB59 (cm^R^) in *S. aureus* SA502a (*agr* type II *agrP3-gfp* reporter strain) | (Malone et al., 2007) |
| *S. aureus* | AH1747 | KPL2433 | pDB59 (cm^R^) in *S. aureus* MW2 (*agr* type III *agrP3-gfp* reporter strain) | (Kirchdoerfer et al., 2011) |
| *S. aureus* | AH1871 | KPL2434 | pDB59 (cm^R^) in *S. aureus* MN TG(406) (*agr* type IV *agrP3-gfp* reporter strain) | (Kirchdoerfer et al., 2011) |
| *S. aureus* | JE2 | KPL2115 | Plasmid-free derivative of USA300 LAC | (Fey et al., 2013) |
| *S. aureus* | NE514 | KPL2348 | JE2 *sarT*::Tn (erm^R^) | (Fey et al., 2013) |
| *S. aureus* | NE1193 | KPL2346 | JE2 *sarA*::Tn (erm^R^) | (Fey et al., 2013) |
| *S. aureus* | NE1532 | KPL2343 | JE2 *agrA*::Tn (erm^R^) | (Fey et al., 2013) |
| *S. aureus* | NE95 | KPL2344 | JE2 *agrB*::Tn (erm^R^) | (Fey et al., 2013) |
| *S. aureus* | KPL2471 | KPL2471 | JE2 pMR371 | this study |
| *S. aureus* | KPL2514 | KPL2514 | JE2 pMR372 | this study |
| *S. aureus* | KPL2474 | KPL2474 | NE514 pMR371 | this study |
| *S. aureus* | KPL2473 | KPL2473 | NE1193 pMR371 | this study |
| *S. aureus* | KPL2472 | KPL2472 | NE1532 pMR371 | this study |
| *S. aureus* | KPL2389 | KPL2389 | JE2 ∆*spa* | this study |
| *S. aureus* | KPL2496 | KPL2496 | JE2 ∆*spa* pEPSA5 | this study |
| *S. aureus* | KPL2497 | KPL2497 | JE2 ∆*spa* pEPSA5-*spa* | this study |

**S3 Table. Primers used in this study.** Capitalized letters indicate restriction site sequences.

| Primer # | Primer Sequence |
| --- | --- |
| oKL304 | ataGCGGCCGCgttattattatttttgacaccagaccaac |
| oKL305 | ataGTCGACaGGATCCaggaggtttatatgaccatgattac |
| oKL308 | ataGTCGACtatctctattacgcaagtgtgct |
| oKL311 | gacgatccttcagtgagc |
| oKL312 | gccaggcttgttattgtc |
| oKL319 | attGGATCCtgtatgtatttgtaaagtcatcataatata |
| oKL339 | gtaaattagccgattgctct |
| oKL340 | tcacgaccagattttgtaga |
| oKL377 | ttgccaattccaaatactgtgagtga |
| oKL378 | gtcaagcctgaagtcgatatgactataaaa |
| oKL379 | ataGTCGACcgtttaacaacacaagaattattatatc |
| oKL380 | attGGATCCcttaaaatttaaatttgaagataacaaaa |
| oKL554 | ataGTCGACtatctctattacgcaagtgtgct |
| oKL555 | ataGGATCCctatcgttgtgtattgtttgttt |

**Supplemental Materials Figures and Legends**

**Table S1. Transcriptional response of *S. aureus* to cocultivation with *C. striatum*.** These are the 469 *S. aureus* genes that were differentially expressed ≥2-fold in coculture with *C. striatum*. Differential expression was assessed from biological duplicates in co- vs. monoculture and determined by analysis with Rockhopper. ^a^Data are presented as fold change in coculture compared to monoculture. All genes included exhibited differential expression with a significance of *q* <0.01 using a Benjamini-Hochberg correction with a false discovery rate <1%.





**Figure S1. *S. aureus* P*_psmβ1_* promoter activity is decreased in response to *C. striatum*.** In response to cocultivation with *C. striatum*, *S. aureus* JE2 expression of plasmid-borne P*_psmβ1_-lacZ* (pMR372) decreased 5.2-fold as measured in Miller units. Growth conditions were identical to those used for RNAseq analysis. Histogram bars represent the mean of three biological replicates. Error bars represent SEM and **p* < 0.001 by two-tailed Student’s *t* test.


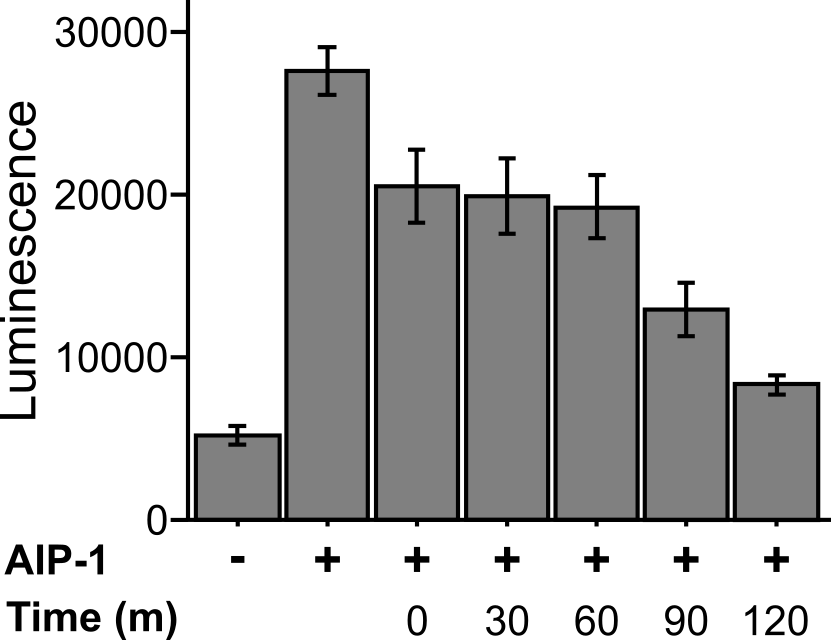


**Figure S2. *C. striatum* CFCM inhibition of AIP-1 signaling is time-dependent.** Using reporter assay conditions identical to those in Fig. 1, we observed that *C. striatum* CFCM diminished *agrP3* reporter activity only after pre-incubation of AIP-1 with CFCM indicating that this process is time-dependent and occurs independently of the reporter strain. An AIP-1-untreated negative control (column 1) shows the background luminescence and an AIP-1-treated positive control (column 2) shows the maximal induction of the reporter strain. *C. striatum* CFCM was incubated with partially purified AIP-1 produced from *E. coli* for the indicated times (columns 3-7) prior to addition to the reporter strain. For each bar, *n*=4 and error bars represent SEM.





**Figure S3. The increase in *S. aureus* P*_spa_* promoter activity in response to *C. striatum* is dependent on *agrA*.** Cocultivation of *C. striatum* with *S. aureus* JE2 results in increased expression of plasmid-borne P*_spa_-lacZ* (pMR371) as measured by comparing the fold change in β-galactosidase activity when *S. aureus* was grown in co- vs. monoculture (WT). This response was absent in an *agrA::Tn* mutant (AgrA^-^). The ratio of P*_spa_* activity was greater in a *sarT::Tn* mutant (SarT^-^) than WT; however, the β-galactosidase activity for SarT^-^ monoculture was 4.4-fold less than WT monoculture. Growth conditions were identical to those used for RNAseq analysis. Histogram bars represent the mean of three biological replicates. Error bars represent SEM and **p* < 0.005 by two-tailed Student’s *t* test with Bonferroni correction for multiple testing.


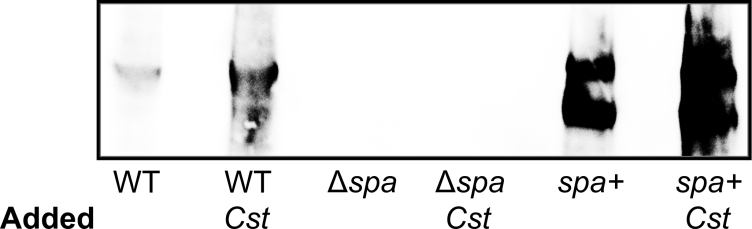


**Figure S4. Exposure to C. *striatum* CFCM increased immunoblot detection of SpA from *S. aureus* strains with an intact *spa* gene.** *S. aureus* strains were grown under conditions identical to those used in Figure 4 (prior to hemolysis measurements). To each lane of the gel, we added 125 ng of total protein from whole cell lysates from each indicated condition. After transfer, the membrane was probed with a 1:15,000 dilution of a rabbit α-SpA HRP conjugated antibody. No SpA detection was evident in the *spa*-deletion mutant (Δ*spa*). SpA band intensity (56 kDa) qualitatively increased in *C. striatum* CFCM-treated wild-type *S. aureus* (WT) and the *spa*-deletion mutant carrying pEPSA5 containing *spa* expressed from its native promoter (*spa*+). A lower molecular weight α-SpA reactive band was also present in the *spa*+ strain under both conditions.





**Figure S5. *S. aureus* exposure to *C. striatum* CFCM results in reduced HL-60 phagocytosis of *S. aureus*.** Human HL-60 phagocytosis of FITC-labeled *S. aureus* cells was quantified by flow cytometry (*n*=3). Exposure of both the wild type (WT) and *spa*-deletion mutant (Δ*spa*) to *C. striatum* CFCM (*Cst*) resulted in a decrease (-21% and -30% respectively) in phagocytosis by differentiated HL-60 phagocytes compared to *S. aureus* strains not exposed to *C. striatum* CFCM. For unexposed *S. aureus* strains, 27% more Δ*spa* cells (medium gray bar) were phagocytosed than wild type (dark gray bar). Error bars represent SEM. Data were analyzed by two-tailed Student’s *t* test with Bonferroni correction for multiple testing (**p* < 0.05).
